# Intrinsic Origins of Crack Generation in Ni-rich LiNi_0.8_Co_0.1_Mn_0.1_O_2_ Layered Oxide Cathode Material

**DOI:** 10.1038/srep39669

**Published:** 2017-01-03

**Authors:** Jin-Myoung Lim, Taesoon Hwang, Duho Kim, Min-Sik Park, Kyeongjae Cho, Maenghyo Cho

**Affiliations:** 1 Department of Mechanical and Aerospace Engineering, Seoul National University, 1 Gwanak-ro, Gwanak-gu, Seoul 08826, Republic of Korea; 2 Department of Advanced Materials Engineering for Information and Electronics, Kyung Hee University, 1732 Deogyeong-daero, Giheung-gu, Yongin 17104, Republic of Korea; 3 Department of Materials Science and Engineering and Department of Physics, University of Texas at Dallas, Richardson, TX 75080, USA

## Abstract

Ni-rich LiNi_0.8_Co_0.1_Mn_0.1_O_2_ layered oxide cathodes have been highlighted for large-scale energy applications due to their high energy density. Although its specific capacity is enhanced at higher voltages as Ni ratio increases, its structural degradation due to phase transformations and lattice distortions during cycling becomes severe. For these reasons, we focused on the origins of crack generation from phase transformations and structural distortions in Ni-rich LiNi_0.8_Co_0.1_Mn_0.1_O_2_ using multiscale approaches, from first-principles to meso-scale phase-field model. Atomic-scale structure analysis demonstrated that opposite changes in the lattice parameters are observed until the inverse Li content *x* = 0.75; then, structure collapses due to complete extraction of Li from between transition metal layers. Combined-phase investigations represent the highest phase barrier and steepest chemical potential after *x* = 0.75, leading to phase transformations to highly Li-deficient phases with an inactive character. Abrupt phase transformations with heterogeneous structural collapse after *x* = 0.81 (~220 mAh g^−1^) were identified in the nanodomain. Further, meso-scale strain distributions show around 5% of anisotropic contraction with lower critical energy release rates, which cause not only micro-crack generations of secondary particles on the interfaces between the contracted primary particles, but also mechanical instability of primary particles from heterogeneous strain changes.

Ni-rich transition metal layered oxides (LiNi_*x*_Co_*y*_Mn_1−*x*−*y*_O_2_, with *x* > 0.5, Ni-rich NCM) have been spotlighted during the past decade as the most promising candidates for high capacity cathode materials in Li-ion batteries (LIBs) due to their high energy densities (>200 mAh g^−1^ until ~4.6 V *vs*. Li/Li^+^)^1^,^2^,^3^. Although LiNi_1/3_Co_1/3_Mn_1/3_O_2_ (NCM111) has been successfully commercialized, in order to meet the demand for large-scale energy storage applications such as electric vehicles (EVs) and energy storage systems (ESSs), further NCM research and development have been directed toward enhancing its specific capacity by increasing the ratio of the Ni component toward LiNi_0.8_Co_0.1_Mn_0.1_O_2_ (NCM811)^4^,^5^,^6^.

While the increase of the Ni ratio in NCM contributes to an enhanced specific discharge capacity, it also results in severe capacity degradation caused by cation mixing, surface side reactions, and crack propagation with structural instability^1^. To better understand these challenges, Jung *et al*. investigated the degradation mechanism of the phase transformation induced by cation mixing from the surface to bulk using *ex situ* structural analysis^7^. Similarly, Lin *et al*. described the surface reconstruction and chemical evolution of the rhombohedral layered structure to a cubic spinel structure using high-throughput X-ray absorption spectroscopy^8^. As theoretical approaches, electronic correlations for the redox reactions between the multivalent transition metals in the Ni-rich NCM^9^ and stability analysis with respect to the various ratios of the Ni, Co, and Mn components in the NCM^10^ have been performed through first-principles calculations. On the bases of these fundamental data, many researchers have suggested solutions to resolve the cyclic degradation problem. Along with diverse approaches such as morphology control^11^, elemental doping^12^,^13^,^14^,^15^ and surface coating^16^,^17^,^18^,^19^,^20^, Sun *et al*. have suggested various effective ways to reduce cyclic degradation and improve electrochemical performance through the design of core-shell^21^, gradient core-shell^3^,^22^, and full concentration gradient structures^2^,^23^,^24^ for Ni-rich NCM cathodes.

Nevertheless, micro-crack propagation in the secondary particle related to structural instability remains problematic, although cation mixing and surface deterioration can be prevented through the above-mentioned approaches. Among the promising Ni-rich compounds, severe structural changes and crack propagation have been observed experimentally in LiNi_*x*_Co_*y*_Al_1−*x*−*y*_O_2_ (NCA) cathodes^25^,^26^,^27^,^28^. Although, Meng *et al*. recently reported the experimental observation of micro-crack generation of NCM811 with severe cyclic degradation and suggested surface coating with Li_2_TiO_3_ to mitigate the crack propagation^29^, the fundamental origin has not been adequately addressed for the Ni-rich NCM cathodes^1^. Moreover, the underlying mechanism for the phase transformation and structural changes in NCM811 is not fully understood. For these reasons, we focused on the study of intrinsic characteristics of the phase transformation and structural changes of NCM811 to elucidate its inherent structural instability regardless of cation mixing and surface deterioration.

Through multiscale phase-transformation mechanics based on first-principles calculations, here, we investigated the fundamental reaction mechanism, structural distortions, thermodynamic combined-phase (CP) behaviours, and meso-scale phase-separation kinetics for NCM811 with respect to varying Li concentration. Since the redox reactions during cycling mainly involve the Ni and O ions, the Co and Mn ions would retain similar electronic structures before and after delithiation. Anisotropic structural changes are observed between the *ab* plane and *c* axis, which result in anisotropic shrinkage of the entire structure. In particular, an abrupt collapse of the structure is observed for an inverse Li content *x* = 0.75–1.0 in Li_1−*x*_Ni_0.8_Co_0.1_Mn_0.1_O_2_. The CP behaviour shows generally one-phase reactions with lower phase barriers below *x* = 0.75. However, a two-phase region with a remarkably higher phase barrier is presented after *x* = 0.75 and inactive phases are formed, which not only agrees well with the experiments of Ohzuku *et al*^30^. but also correlates with the collapse of the structure after *x* = 0.75. Based on these first-principles calculation results, meso-scale phase separation simulations were performed, which reveals heterogeneous phase transformations and structural changes at different Li concentrations. Therefore, an intrinsic limitation of NCM811 exists in the region *x* = 0.75–0.81 (~220 mAh g^−1^) due to the inactive phase separation and abrupt structural changes. Around 5% of the anisotropic contraction was observed in the nanodomain, which induces the contractions of the primary particles. In addition, very low critical energy release rates for crack generation of fully-lithiated and delithiated NCM811 were calculated. Such large volume reduction and low critical energy release rate could be the reason of causing the micro-crack generations in the secondary particles on the interfaces between the contracted primary particles. Further, the heterogeneous strain changes cause severe mechanical instability within the primary particles, which could be the reason of the generation of nano-cracks as a seed of the micro-cracks. These findings should provide helpful insights for the development of Ni-rich NCM cathode materials in the Li-ion battery research community.

## Results and Discussion

### Redox Mechanism and Structural distortion

**Figure 1:**
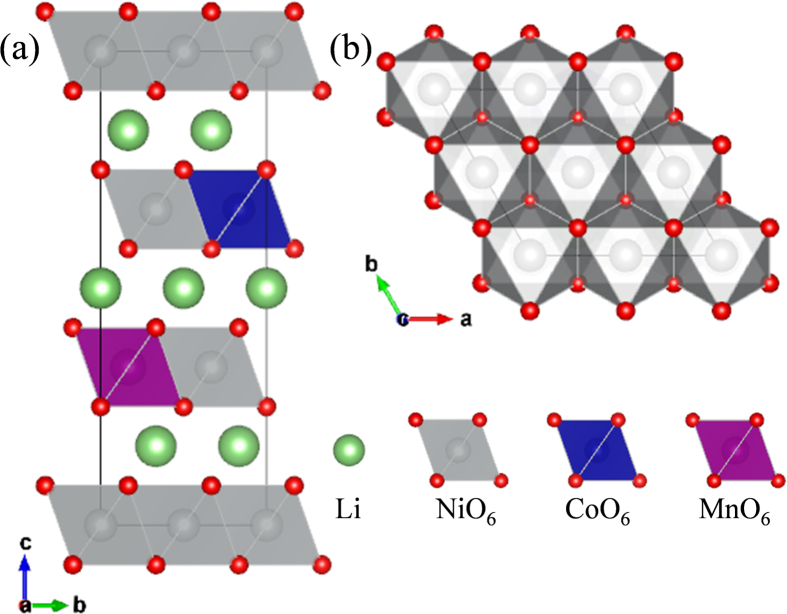
Atomic models of Ni-rich LiNi_0.8_Co_0.1_Mn_0.1_O_2_ (NCM811) layered oxide (*R*-3*m*) projected onto the (**a**) *bc* and (**b**) *ab* planes.

Figure 1a and b show the atomic model used for the first-principles calculations projected on the *bc* and *ab* planes, respectively. The atomic model was developed based on the rhombohedral layered oxide structure (*R-*3*m*) of LiNiO_2_ (Inorganic Crystal Structure Database (ICSD) ID: 10499). To represent the stoichiometry of NCM811, supercells of 2 × 2 × 1 with 12*f.u.* were used; the exact stoichiometry of the supercell in this study is LiNi_0.8333_Co_0.0833_Mn_0.0833_O_2_ (Li_12_Ni_10_Co_1_Mn_1_O_24_). The model was developed using high-throughput calculations based on previously described schemes^31^,^32^,^33^,^34^,^35^. As shown in Fig. 1a, Co and Mn are located separately in the Ni-rich environment of the layered oxide structure, which would be thermodynamically related to *d*-electronic stability of the crystal field^36^,^37^.

**Figure 2:**
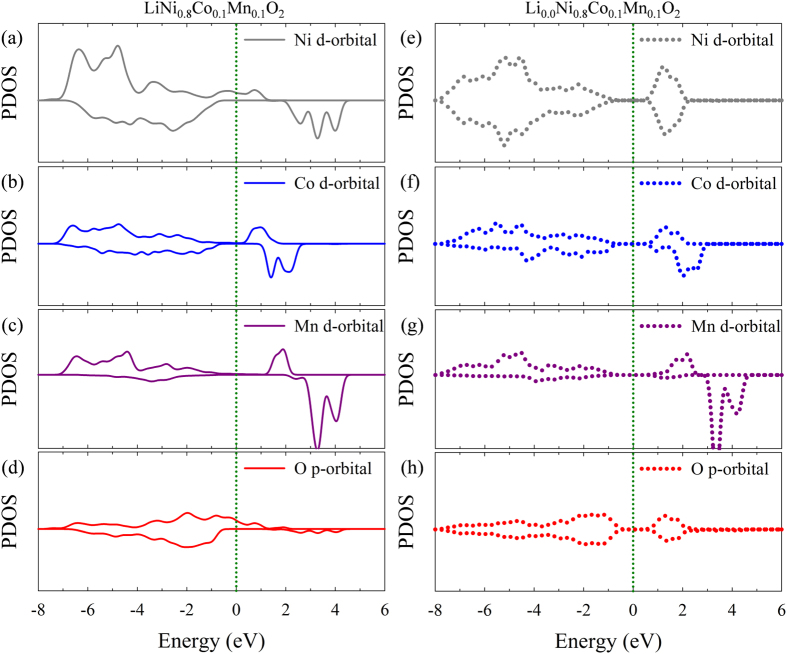
(**a,b,c,d**) Partial density of states (PDOSs) of (a) Ni *d*- (gray solid line), (**b**) Co *d*- (blue solid line), (**c**) Mn *d*- (purple solid line), and (**d**) O *p*- (red solid line) orbitals in LiNi_0.8_Co_0.1_Mn_0.1_O_2_. (**e,f,g,h**) PDOSs of (**e**) Ni *d*- (gray dashed line), (f) Co *d*- (blue dashed line), (**g**) Mn *d*- (purple dashed line), and (h) O *p*- (red dashed line) orbitals in Li_0.0_Ni_0.8_Co_0.1_Mn_0.1_O_2_. The Fermi level is 0.0 eV (green dotted line).

Figure 2 presents the partial (projected) density of states (PDOSs) of the Ni, Co, and Mn *d*-orbitals and the O *p*-orbitals in Li_1_Ni_0.8_Co_0.1_Mn_0.1_O_2_ and Li_0_Ni_0.8_Co_0.1_Mn_0.1_O_2_ (Fig. 2a–d and 2e–h, respectively). In the fully lithiated state (Li_1_Ni_0.8_Co_0.1_Mn_0.1_O_2_), the Ni, Co, and Mn *d*-orbitals are shown as Ni^3^-like, Co^3+^, and Mn^4+^-like shapes due to the effect of the *d*-electronic donor^9^,^38^, in agreement with a previous report^10^. The thermodynamic stability of the Co^3+^ and Mn^4+^ crystal fields could be satisfied by maintaining their separate locations. Based on the electronic structures, further, the redox reactions of NCM811 can be understood. Fig. 2e–h present the PDOSs of the fully delithiated state (Li_0_Ni_0.8_Co_0.1_Mn_0.1_O_2_). The charts reveal Ni^4+^, Co^3+^, and Mn^4+^-like shapes, which indicate that the cationic redox reaction of Ni^3+^-like to Ni^4+^ is a major cationic redox process. Additionally, Fig. 2d and 2h indicate a notable anionic redox reaction contribution from O ions. Therefore, the superior electrochemical performance of the Ni-rich NCM can be attributed to the combination of the anionic redox reactions^39^,^40^ and the cationic redox reactions of the Ni ions in the stable crystal fields of the Co and Mn *d*-orbitals.

**Figure 3:**
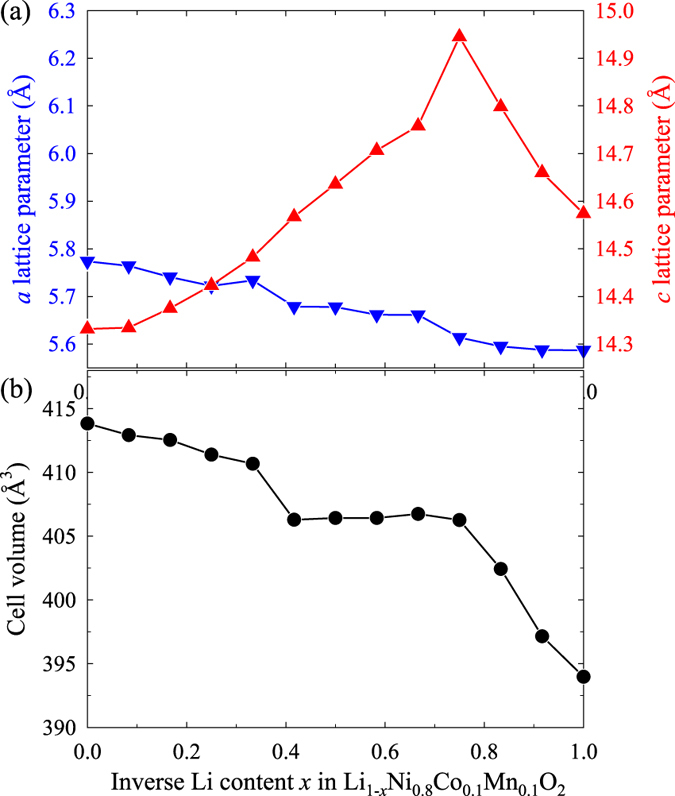
(**a**) Calculated *a* (blue inverse triangles and solid line, left axis) and *c* lattice parameters (red triangles and solid line, right axis), and (**b**) volume of cell (black circles and solid line) with respect to the inverse Li content *x* in Li_1−*x*_Ni_0.8_Co_0.1_Mn_0.1_O_2_.

From a structural point of view, NCM811 suffers from severe structural changes with anisotropic distortions. Figure 3a shows the expansion and contraction of the *a* (blue left axis) and *c* (red right axis) lattice parameters as a function of the inverse Li content *x* in Li_1−*x*_Ni_0.8_Co_0.1_Mn_0.1_O_2_. In the *a* direction, the lattice decreases gradually throughout the reaction from *x* = 0 to 1, whereas the lattice in the *c* direction shows non-monotonic behaviour, increasing from *x* = 0.0 to 0.75 and decreasing thereafter. In other words, the lattice parameters change in opposite directions from *x* = 0.0 to 0.75, but in the same direction thereafter. However, after *x* = 0.75, structural collapse is observed due to the rapid volume reduction, as shown in Fig. 3b, which is caused by the complete extraction of Li ions from between the transition metal layers. Therefore, the structural changes of NCM811 during electrochemical reactions are likely to be harmful to the cyclic performance due to the lattice distortions in opposing directions (0.0 < *x* < 0.75) and the volumetric collapse (0.75 < *x* < 1.0).

### Heterogeneous Phase Transformation

**Figure 4:**
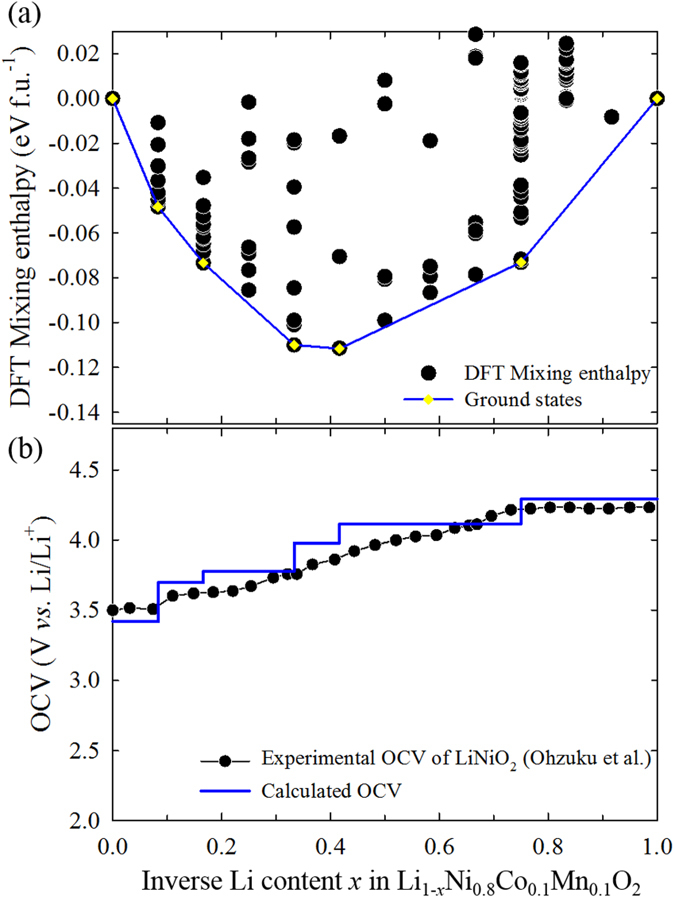
(**a**) Mixing enthalpies from density functional theory (DFT) calculations (black circles) with ground states (yellow diamonds and blue line). (**b**) Open-circuit voltage (OCV) calculated using first principles (blue solid line) and reproduced by experiment (black circles and line) from Ohzuku *et al*^30^. versus inverse Li content *x* in Li_1−*x*_Ni_0.8_Co_0.1_Mn_0.1_O_2_.

To understand the thermodynamic phase transformations, the phase behaviours were investigated by calculating the DFT mixing enthalpies *H*_*DFT*_(*x*) with respect to normalized inverse Li content *x* using Eq. (1), as presented in Fig. 4a. Seven ground states are observed, including the initial (*x* = 0) and final (*x* = 1) states. The CP redox reaction of NCM811 consists of two one-phase reactions (0.0 < *x* < 0.1667; 0.3333 < *x* < 0.4167) and three two-phase reactions (0.1667 < *x* < 0.3333; 0.4167 < *x* < 0.75; 0.75 < *x* < 1.0). To predict the electrochemical behaviour, Fig. 4b presents the OCVs based on the ground states in Fig. 4a; the shape of the curve is similar to the experimental OCV values for LiNiO_2_ estimated previously by the galvanostatic intermittent titration technique (GITT)^30^.

**Figure 5:**
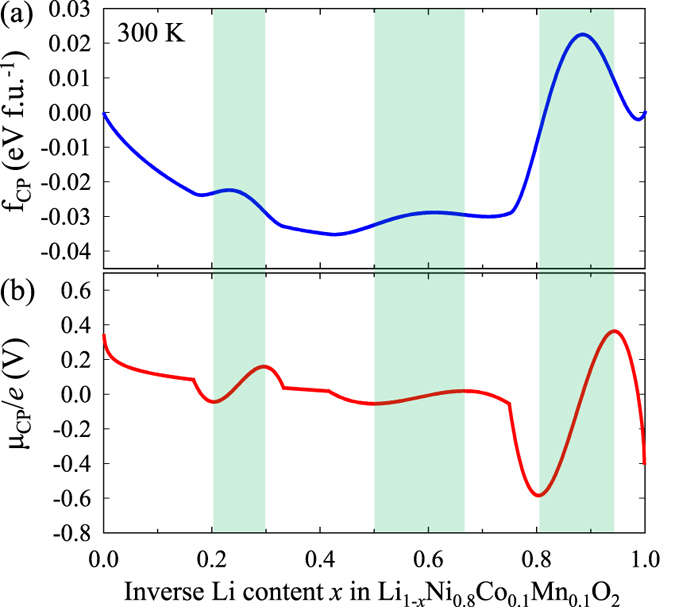
(**a**) Combined-phase (CP) free energy (blue solid line) and (**b**) CP chemical potential (red solid line) at room temperature (300 K) with spinodal regions (green shaded areas) with respect to inverse Li content *x* in Li_1-*x*_Ni_0.8_Co_0.1_Mn_0.1_O_2_.

By considering the configurational entropy at a finite temperature (300 K), more general phase behaviours can be understood using the CP free energy *f*_*CP*_(*x*) and the CP chemical potential *μ*_*CP*_ of Eqs (2) and (3), as shown in Fig. 5. Spinodal regions, where two-phase reactions occur, are indicated as green shaded regions in Fig. 5. Figure 5a shows that the first and second phase barriers from *x* = 0.21 to 0.30 and from *x* = 0.50 to 0.67 are low, but the third phase barrier from *x* = 0.81 to 0.93 is remarkably steep and higher than the others. This means that phase separations are likely to be impeded due to the low phase barriers and the low slopes of the chemical potentials before *x* = 0.75, such that relatively smooth reactions similar to the one-phase reaction can be generated, rather than the two-phase reaction. In contrast, after *x* = 0.75, rapid phase separation by a two-phase reaction is likely to occur as indicated by the higher phase barrier in Fig. 5a and the sharp slope of the chemical potential in Fig. 5b. More importantly, the strong phase separation after *x* = 0.75 results in a highly Li-deficient phase (Li_0_Ni_0.8_Co_0.1_Mn_0.1_O_2_), leading to severe structural transformations to inactive phases such as the NiO rock salt phase previously observed by *ex situ* transmission electron microscopy in Ni-rich NCM^7^.

### Origins of Crack Generation

**Figure 6:**
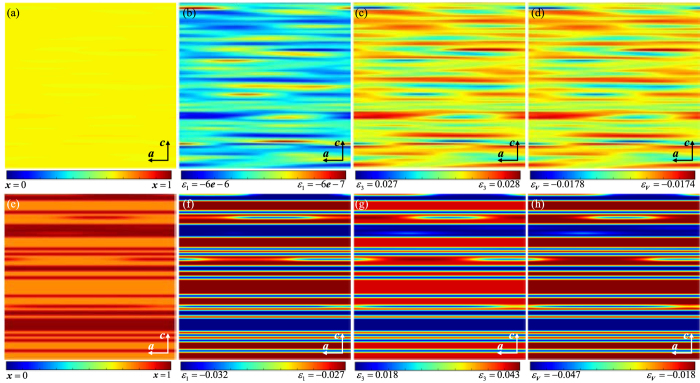
Meso-scale phase transformations in the nanodomain with dimensions of 31.36 × 31.36 nm in Li_1−x_Ni_0.8_Co_0.1_Mn_0.1_O_2_ at room temperature (300 K) from a solid solution of inverse Li concentration x = 0.65 (**a,b,c,d**) and 0.85 (**e,f,g,h**) at dimensionless time 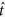 = 5. (**a,e**), (**b,f**), (**c,g**), and (**d,h**) are the distributions on the *ac* plane of the inverse Li concentration *x*, strain in *a* direction *ε*_1_, strain in *c* direction *ε*_3_, and volumetric strain *ε*_*V*_, respectively.

To describe the meso-scale phase transformations of LiNi_0.8_Co_0.1_Mn_0.1_O_2_ in a nanodomain with dimensions of 31.36 nm × 31.36 nm at 300 K, a phase-field model analysis was conducted by solving Eqs (9) and (10). As simulation parameters, we set *D* as 10^−8^ cm^2^ s^−1^ based on an experimental value^41^, *λ* as 0.49 nm from the interlayer distance of the atomic model, and the 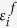 values as 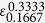 = 128.66 eV, 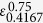 = 7.85 eV, and 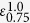 = 169.95 eV. The phase transformation simulations were carried out on the *ac* plane during relaxation from the solid solution at *x* = 0.65 (Fig. 6a) with a theoretical charge capacity of ~179 mAh g^−1^ and *x* = 0.85 (Fig. 6e) with a theoretical charge capacity of ~234 mAh g^−1^. The *a, c*, and volumetric strains (denoted as *ε*_1_, *ε*_3_, and *ε*_*V*_, respectively) are displayed in Fig. 6b (x = 0.65) and f (x = 0.85), 6c (x = 0.65) and 6g(x = 0.85), and 6d (x = 0.65) and 6h(x = 0.85), respectively. The strains are defined as *ε*_1_ = Δ***a***/***a***_*x = *0_, *ε*_3_ = Δ***c***/***c***_*x=*0_, and *ε*_*V*_ = Δ***V***/***V***_*x*=0_, where *a* and *c* are the lattice parameters and *V* is the volume, respectively. Finally, 2D diffusion in the *a* and *b* directions was applied, and the phase separation was triggered by random noise.

From the distribution of the inverse Li concentration, distinct phase separation is observed in Fig. 6e at *x* = 0.85, correlated with the sharp slope of the chemical potential in Fig. 5b, but only slight phase separation is observed in Fig. 6a at *x* = 0.65. For this reason, smooth electrochemical reactions may be possible until *x* = 0.65 (~179 mAh g^−1^), but phase separation to an inactive phase (Li_0_Ni_0.8_Co_0.1_Mn_0.1_O_2_) should occur at *x* = 0.85 (~234 mAh g^−1^), meaning that the third spinodal region from *x* = 0.81 to 0.93 induces intrinsically irreversible characteristics. Furthermore, from a structural perspective, at *x* = 0.65, not only are contraction strains of *ε*_1_ and *ε*_3_ observed, but also slight differences occur between *ε*_1_ (–6 × 10^−6^ < *ε*_1_ < –6 × 10^−7^) and *ε*_3_ (0.027 < *ε*_3_ < 0.028), as shown in Fig. 6b and c. Fortunately, the extent of structural change can be considered as reasonably small; e.g., the difference in *ε*_*V*_ is less than 4 × 10^−4^, as illustrated in Fig. 6d. Severe structural distortions occur at *x* = 0.85. All the absolute changes in the three strains at *x* = 0.85 are remarkably larger than those at *x* = 0.65: *ε*_1_ changes relatively slightly (–0.032 < *ε*_1_ < –0.027), whereas *ε*_3_ and *ε*_*V*_ vary by as much as 5% (0.018 < *ε*_3_ < 0.043, –0.047 < *ε*_*V*_ < –0.018) (Fig. 6f,g and h, respectively). Videos attached in the supplementary information illustrate the specific evolution of the inverse concentration of Li and the volumetric strain.

**Figure 7:**
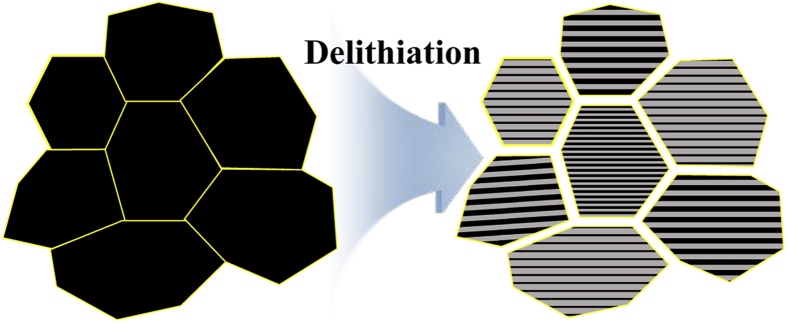
Schematic illustration of micro-crack generations in secondary particles on the interfaces of the contracted primary particles during delithiation.

In the meso-scale phase transformation phenomena, the structural distortions are heterogeneously generated in the nanodomain due to the blocking of diffusion in the *c* direction and the rapid phase separation, which is shown in the apparent red and blue regions of Fig. 6f,g and h. Therefore, the structural changes in NCM811 occur more severely in the third spinodal region, and, combined with the abrupt phase separation, limit the intrinsic specific capacity to less than 220 mAh g^−1^ for *x* = 0.81. As shown in Fig. 7, the around 5% of anisotropic contraction and the ~3.9% of average contraction (Fig. 6h) in the nanodomain would cause the contraction of each primary particle, and then the gaps between the primary particles would result in the micro-crack generations in the secondary particles on the interfaces between the primary particles, which could be the origins and mechanism on the experimental observations of the micro-crack propagations^25^,^26^,^27^,^28^,^29^. Further, the heterogeneous distribution of the anisotropic strain changes causes the severe mechanical instability of the primary particles that could induce the generation of nano-cracks, which could be a crack opening of the micro-cracks.

In addition, Table 1 shows critical energy release rates of Li_1_Ni_0.8_Co_0.1_Mn_0.1_O_2_ and Li_0_Ni_0.8_Co_0.1_Mn_0.1_O_2_ calculated by DFT, which describe the critical energy for the crack generation and are related to the surface energy γ for brittle materials (*G*_*c*_ = 2γ) based on Griffith’s theory. Though fully-lithiated NCM811 has low *G*_*c*_ (2.4308 J m^−2^), the delithiated NCM811 has even lower *G*_*c*_ (−0.0064 J m^−2^), which would result from the structural instability between layers at lower Li concentrations. In other words, not only NCM811 is fragile to crack generation, but also it gets weakened at delithiated states, which means the 5% of anisotropic contraction could be enough to generate crack propagations.

Recently, Meng *et al*. reported that the severe crack generation in NCM811 particles induces a significant performance degradation^29^. According to the scanning electron microscopy (SEM) observation, particle fractures and fragmentation of NCM811 particles were evident after cycles. Due to this crack generation, the discharge capacity of NCM811 was remarkably decreased with increasing overpotentials during cycles. From our fundamental understanding, we suggest that the origin of crack generation is the contraction of primary particles with a mechanical instability caused by heterogeneous phase transformation and anisotropic strain changes. In addition, the lower *G*_*c*_ at delithiated states contributes to a severe crack propagation. Finally, it is expected that these could be resolved by reducing the inhomogeneity and anisotropy of structural changes and increasing *G*_*c*_.

## Conclusion

The intrinsic limitations of a Ni-rich NCM811 cathode material were investigated in terms of its phase transformations and structural distortions using multiscale approaches combining first-principles calculations and the CP phase-field model. The major redox mechanism of NCM811 was determined as a combination of the cationic redox reactions of Ni with the anionic redox reactions of O. The atomic-scale structural analysis showed that opposite lattice changes are generated until *x* = 0.75, followed thereafter by the gradual decrease all of the lattice parameters due to the collapse of the transition metal layers. The CP behaviours represent the three two-phase reaction regions, wherein the third exhibits a higher phase barrier and a sharp rise in the chemical potential. This causes rapid phase separation, forming an inactive and highly Li-deficient phase. In the meso-scale phase transformation, heterogeneous phase separations are observed, and severe phase transformation occurs near the third spinodal region at *x* = 0.85. Further, the ~3.9% of the average contraction and lower critical energy release rates including the heterogeneous distribution of the anisotropic strain changes observed by the meso-scale strain distributions could induce not only the nano-cracks in the nanodomain of the primary particles from the severe mechanical instability, but also the micro-crack generations on the interfaces between the contracted primary particles. Thus, the combination of the abrupt transformations to inactive phases with the heterogeneous collapse of the layered structure limits the intrinsic performance of NCM811. These findings may help to predict the maximum performance of Ni-rich NCM cathodes, and the mechanistic insights on the phase transformations and structural distortions could provide clues toward improving cathode materials for battery applications.

## Methodology

### First-principles calculations

For the atomic-scale simulations, first-principles calculations were conducted using spin-polarized density functional theory (DFT) with the generalized gradient approximation (GGA) according to the Perdew-Wang 91 exchange-correlation functional^42^. The Vienna *Ab Initio* Simulation Package (VASP) was utilized to implement a plane-wave basis set and the projector-augmented wave (PAW) method^43^,^44^. To evaluate accurate electrochemical and *d*-orbital electronic properties, the Hubbard *U* parameter was used^45^. The *U* values for Ni, Co, and Mn (6.7, 4.91, and 4.64, respectively) were chosen from previous reports^37^,^46^. As computational parameters, a plane-wave cut-off energy of 500 eV and *k*-point meshes of 4 × 4 × 2 for the Brillouin zone sampling were determined by a convergence test. The atomic model for NCM811 was developed by the 12 formula units (*f.u*.) of LiNi_0.8333_Co_0.0833_Mn_0.0833_O_2_ using high-throughput calculations^31^,^32^,^33^,^34^,^35^. All the calculations were based on fully relaxed structures.

### Combined-phase (CP) phase transformation mechanics

For the multiscale analyses from the atomic- to meso-scale, we adopted the CP transformation model^47^,^48^ and the Cahn-Hilliard equation^49^.

First, the DFT mixing enthalpy *H*_*DFT*_(*x*) with respect to the normalized inverse Li content *x* from 0.0 to 1.0 for battery electrodes can be defined as follows:


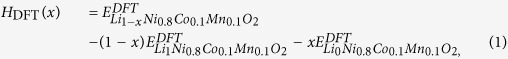


where 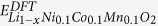 represents the total energy of Li_1−*x*_Ni_0.8_Co_0.1_Mn_0.1_O_2_ as calculated from DFT calculations. From the convex hull analysis of *H*_*DFT*_(*x*), the CP free energy *f*_*CP*_(*x*) and the CP chemical potential *μ*_*CP*_ at finite temperature by considering the configurational entropy^50^ can be determined based on the CP mixing enthalpy *H*_*CP*_(*x*) as follows:


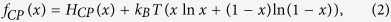



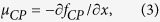



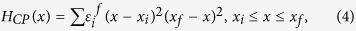


where, *k*_*B*_ and *T* represent the Boltzmann constant and absolute temperature, respectively. Furthermore, the enthalpy coefficient 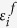 in the reaction regions from *x*_*i*_ to *x*_*f*_ is obtained by parameterization from the CP mixing enthalpy 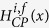 as follows:


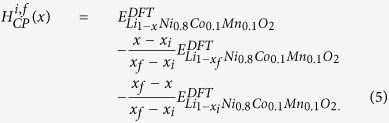


The open-circuit voltage (OCV) *V*_*DFT*_ was calculated using DFT at 0 K as follows:


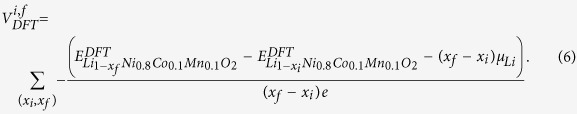


To solve the meso-scale phase transformation phenomena, the CP Cahn-Hilliard energy function *G*_*CP*_ was defined as follows:


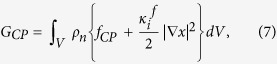


where, *ρ*_*n*_ represents the number of sites per volume *V* and 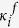 is the gradient energy coefficient determined by the characteristic length *λ* and enthalpy coefficient 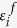 as follows:^51^,^52^


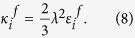


We applied the semi-implicit Fourier-spectral method^53^ with a periodic boundary condition (PBC) to compute the Cahn-Hilliard equation^49^. For non-dimensionalization, *k*_*B*_*T, λ*, and *D*/*λ*_2_ were used as the dimensionless energy, length, and time scales with the use of the diffusion coefficient *D*, respectively. Based on these dimensionless parameters, the Cahn-Hilliard equation can be written as follows:


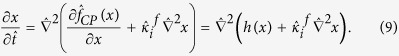


Then, Eq. (9) can be re-written on the Fourier space with a wave vector *k* as follows:


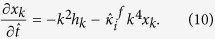


## Additional Information

**How to cite this article**: Lim, J.-M. *et al*. Intrinsic Origins of Crack Generation in Ni-rich LiNi_0.8_Co_0.1_Mn_0.1_O_2_ layered oxide cathode material. *Sci. Rep.* **7**, 39669; doi: 10.1038/srep39669 (2017).

**Publisher's note:** Springer Nature remains neutral with regard to jurisdictional claims in published maps and institutional affiliations.

## Supplementary Material

Supplementary Information

Supplementary Video 1

Supplementary Video 2

Supplementary Video 3

Supplementary Video 4

## Figures and Tables

**Table 1:** Critical energy release rates *G*_*c*_ for a crack generation of Li_1_Ni_0.8_Co_0.1_Mn_0.1_O_2_ and Li_0_Ni_0.8_Co_0.1_Mn_0.1_O_2_ based on Griffith’s theory.

	Li_1_Ni_0.8_Co_0.1_Mn_0.1_O_2_	Li_0_Ni_0.8_Co_0.1_Mn_0.1_O_2_
*G*_*c*_	2.4308 J m^−2^	−0.0064 J m^−2^
