# Supplementary material for: Intrinsic Origins of Crack Generation in Ni-rich LiNi0.8Co0.1Mn0.1O2 Layered Oxide Cathode Material
Source: Sci Rep. 2017 Jan 3;7:39669. doi: 10.1038/srep39669 (PMC5206713; doi:10.1038/srep39669)
Supplement: Supplementary Information [file srep39669-s1.doc]

*Supplementary Information for*

Intrinsic Origins of Crack Generation in Ni-rich LiNi_0.8_Co_0.1_Mn_0.1_O_2_ Layered Oxide Cathode Material

Jin-Myoung Lim,^1^ Taesoon Hwang,^1^ Duho Kim,^1^ Min-Sik Park,^2^ Kyeongjae Cho,^1,3^ Maenghyo Cho^1^,*

^1^ Department of Mechanical and Aerospace Engineering, Seoul National University, 1 Gwanak-ro, Gwanak-gu, Seoul 08826, Republic of Korea.

^2^ Department of Advanced Materials Engineering for Information and Electronics, Kyung Hee University, 1732 Deogyeong-daero, Giheung-gu, Yongin 17104, Republic of Korea.

^3^ Department of Materials Science and Engineering and Department of Physics, University of Texas at Dallas, Richardson, TX 75080, USA.

Corresponding Author

* E-mail: mhcho@snu.ac.kr

* Video Legends

Video name: Video 1.avi

Video title: concentration (*x* = 0.65)

Distribution of inverse Li concentration *x* on the *ac* plane with respect to the dimensionless time $\hat{t}$ at room temperature (300K) from a solid solution of *x* = 0.65 (Li_0.35_Ni_0.8_Co_0.1_Mn_0.1_O_2_).

Video name: Video 2.avi

Video title: volumetric strain (*x* = 0.65)

Distribution of volumetric strain $\varepsilon_{V}$ on the *ac* plane with respect to the dimensionless time $\hat{t}$ at room temperature (300K) from a solid solution of *x* = 0.65 (Li_0.35_Ni_0.8_Co_0.1_Mn_0.1_O_2_).

Video name: Video 3.avi

Video title: concentration (*x* = 0.85)

Distribution of inverse Li concentration *x* on the *ac* plane with respect to the dimensionless time $\hat{t}$ at room temperature (300K) from a solid solution of *x* = 0.85 (Li_0.15_Ni_0.8_Co_0.1_Mn_0.1_O_2_).

Video name: Video 4.avi

Video title: volumetric strain (*x* = 0.85)

Distribution of volumetric strain $\varepsilon_{V}$ on the *ac* plane with respect to the dimensionless time $\hat{t}$ at room temperature (300K) from a solid solution of *x* = 0.85 (Li_0.15_Ni_0.8_Co_0.1_Mn_0.1_O_2_).
